# Review of Ongoing Activities and Challenges to Improve the Care of Patients With Type 2 Diabetes Across Africa and the Implications for the Future

**DOI:** 10.3389/fphar.2020.00108

**Published:** 2020-03-20

**Authors:** Brian Godman, Debashis Basu, Yogan Pillay, Julius C. Mwita, Godfrey Mutashambara Rwegerera, Bene D. Anand Paramadhas, Celda Tiroyakgosi, Patrick Mbah Okwen, Loveline Lum Niba, Justice Nonvignon, Israel Sefah, Margaret Oluka, Anastasia N. Guantai, Dan Kibuule, Francis Kalemeera, Mwangana Mubita, Joseph Fadare, Olayinka O. Ogunleye, Larry A. Distiller, Enos M. Rampamba, Jeffrey Wing, Debjani Mueller, Abubakr Alfadl, Adefolarin A. Amu, Zinhle Matsebula, Aubrey Kalungia, Trust Zaranyika, Nyasha Masuka, Janney Wale, Ruaraidh Hill, Amanj Kurdi, Angela Timoney, Stephen Campbell, Johanna C. Meyer

**Affiliations:** ^1^Strathclyde Institute of Pharmacy and Biomedical Sciences, University of Strathclyde, Glasgow, United Kingdom; ^2^Division of Public Health Pharmacy and Management, School of Pharmacy, Sefako Makgatho Health Sciences University, Pretoria, South Africa; ^3^Division of Clinical Pharmacology, Karolinska Institute, Karolinska University Hospital Huddinge, Stockholm, Sweden; ^4^Health Economics Centre, University of Liverpool Management School, Liverpool, United Kingdom; ^5^Department of Public Health Medicine, Steve Biko Academic Hospital, University of Pretoria, Pretoria, South Africa; ^6^HIV & AIDS, TB and Maternal, Child and Women’s Health, National Department of Health, Pretoria, South Africa; ^7^Department of Internal Medicine, Faculty of Medicine, University of Botswana, Gaborone, Botswana; ^8^Department of Medicine, Princess Marina Hospital, Gaborone, Botswana; ^9^Department of Pharmacy, Nyangabgwe Hospital, Francistown, Botswana; ^10^Botswana Essential Drugs Action Program, Ministry of Health and Wellness, Gaborone, Botswana; ^11^Effective Basic Services (eBASE) Africa, Bamenda, Cameroon; ^12^Faculty of Health and Medical Sciences, University of Adelaide, Adelaide, SA, Australia; ^13^Department of Public Health, University of Bamenda, Bambili, Cameroon; ^14^School of Public Health, University of Ghana, Legon, Ghana; ^15^Department of Pharmacy, Keta Municipal Hospital, Ghana Health Service, Keta, Ghana; ^16^Department of Pharmacology and Pharmacognosy, School of Pharmacy, University of Nairobi, Nairobi, Kenya; ^17^Department of Pharmacy Practice and Policy, Faculty of Health Sciences, University of Namibia, Windhoek, Namibia; ^18^Department of Pharmacology and Therapeutics, Ekiti State University, Ado-Ekiti, Nigeria; ^19^Department of Medicine, Ekiti State University Teaching Hospital, Ado-Ekiti, Nigeria; ^20^Department of Pharmacology, Therapeutics and Toxicology, Lagos State University College of Medicine, Lagos, Nigeria; ^21^Department of Medicine, Lagos State University Teaching Hospital, Lagos, Nigeria; ^22^Centre for Diabetes & Endocrinology (Pty) Ltd, Johannesburg, South Africa; ^23^Department of Pharmacy, Tshilidzini Regional Hospital, Limpopo Department Of Health, Shayandima, South Africa; ^24^School of Clinical Medicine, University of the Witwatersrand, Johannesburg, South Africa; ^25^Charlotte Maxeke Medical Research Cluster, Johannesburg, South Africa; ^26^National Medicines Board, Federal Ministry of Health, Khartoum, Sudan; ^27^Unaizah College of Pharmacy, Qassim University, Unaizah, Saudi Arabia; ^28^Eswatini Medical Christian University, Mbabane, Swaziland; ^29^Raleigh Fitkin Memorial Hospital, Manzini, Swaziland; ^30^Department of Pharmacy, School of Health Sciences, University of Zambia, Lusaka, Zambia; ^31^Department of Medicine, University of Zimbabwe College of Health Sciences, Harare, Zimbabwe; ^32^Independent Health Systems Consultant, Harare, Zimbabwe; ^33^Independent Consumer Advocate, Brunswick, VIC, Australia; ^34^Liverpool Reviews and Implementation Group, Liverpool University, Liverpool, United Kingdom; ^35^Department of Pharmacology, College of Pharmacy, Hawler Medical University, Erbil, Iraq; ^36^NHS Lothian Director of Pharmacy, NHS Lothian, Edinburgh, United Kingdom; ^37^Centre for Primary Care, Division of Population Health, Health Services Research and Primary Care, University of Manchester, Manchester, United Kingdom; ^38^NIHR Greater Manchester Patient Safety Translational Research Centre, School of Health Sciences, University of Manchester, Manchester, United Kingdom

**Keywords:** Type 2 diabetes, Africa, national initiatives, diagnosis, medicines, adherence, patient groups

## Abstract

**Background:**

There has been an appreciable increase in the number of people in Africa with metabolic syndrome and Type 2 diabetes (T2DM) in recent years as a result of a number of factors. Factors include lifestyle changes, urbanisation, and the growing consumption of processed foods coupled with increasing levels of obesity. Currently there are 19 million adults in Africa with diabetes, mainly T2DM (95%), estimated to grow to 47 million people by 2045 unless controlled. This has a considerable impact on morbidity, mortality and costs in the region. There are a number of issues to address to reduce the impact of T2DM including improving detection rates and current access to services alongside addressing issues of adherence to prescribed medicines. There are also high rates of co-morbidities with infectious diseases such as HIV and tuberculosis in patients in Africa with T2DM that require attention.

**Objective:**

Document ongoing activities across Africa to improve the care of patients with T2DM especially around issues of identification, access, and adherence to changing lifestyles and prescribed medicines. In addition, discussing potential ways forward to improve the care of patients with T2DM based on ongoing activities and experiences including addressing key issues associated with co-morbidities with infectious diseases.

**Our Approach:**

Contextualise the findings from a wide range of publications including internet based publications of national approaches coupled with input from senior level government, academic and other professionals from across Africa to provide future guidance.

**Ongoing Activities:**

A number of African countries are actively instigating programmes to improve the care of patients with T2DM starting with improved diagnosis. This recognises the growing burden of non-communicable diseases across Africa, which has been neglected in the past. Planned activities include programmes to improve detection rates and address key issues with diet and lifestyle changes, alongside improving monitoring of care and activities to enhance adherence to prescribed medicines. In addition, addressing potential complexities involving diabetes patients with infectious disease co-morbidities. It is too early to fully assess the impact of such activities,

**Conclusion:**

There are a number of ongoing activities across Africa to improve the management of patients with diabetes including co-morbidities. However, more needs to be done considering the high and growing burden of T2DM in Africa. Ongoing research will help further benefit resource allocation and subsequent care.

## Introduction

The number of overweight and obese people is rising across Africa as a result of changes in lifestyles and reduced physical activity, more sedentary lifestyles, changing dietary habits and easy access to inexpensive processed foods, cultural habits, and increasing urbanization (Owiredu et al., 2008; Mbanya et al., 2014; Cois and Day, 2015; Ofori-Asenso et al., 2016; Werfalli et al., 2016; Buse et al., 2017; Kingdom of Eswatini - Ministry of Health, 2017; Ministry of Health and Social Services Namibia, 2017; SEMDSA, 2017; Werfalli et al., 2016; Zekewos et al., 2018; Osei-Yeboah et al., 2019). Such changes are leading to an increase in the number of patients with metabolic syndrome and type 2 diabetes (T2DM) across the continent (Mbanya et al., 2014; Kharroubi and Darwish, 2015; Agyemang et al., 2016; Todowede and Sartorius, 2017). Studies in South Africa suggest over 50% of the adult population (18 years and above) are overweight or obese and rising (Cois and Day, 2015), and in Nigeria 62% and 49% respectively of adults are overweight or obese (Commodore-Mensah et al., 2014). Kenyan women were also reported to be significantly more likely to have abdominal obesity compared to men (50.2% *vs.* 12.1%) (Mohamed et al., 2018). However, diabetes in sub-Saharan Africa is also present in patients with low to normal body mass indices (BMIs), which may reflect genetic diversities compared with populations in other continents (Kibirige et al., 2019). Overall, diabetes mellitus is a growing problem across Africa with an estimated 19 million adults affected including 14.2 million in sub-Saharan Africa (SSA) (International Diabetes Federation, 2019). Numbers are likely to grow to 41.6 million across Africa by 2045, principally T2DM (International Diabetes Federation, 2019).

As mentioned, rapid urbanization in Africa is one of the principal risk factors for increasing rates of T2DM across the continent. SSA is often regarded as the world's fastest urbanizing region, with the global share of African urban residents projected to grow from 11.3% in 2010 to 20% in 2050 (Saghir and Santoro, 2018). The city of Johannesburg in South Africa has been a part of a world-wide initiative called “Cities Changing Diabetes,” the purpose of which is to improve the management of patients with diabetes in urban areas using the principles of halves, i.e., approximately 50% of people with diabetes are diagnosed, of whom 50% receive care, of whom 50% achieve treatment target (recommended glucose levels), of whom approximately 50% would subsequently lead a life free from diabetes related complications equating to 6% of those with diabetes (Napier et al., 2017).

Activities are just starting across Africa to improve the care of patients with T2DM (**Table 1**). Typically though, the awareness of diabetes among patients remains a major challenge in SSA with only a minority of patients currently aware of their diabetic status and being actively treated, with only a small percentage being diagnosed pre-diabetes (Zekewos et al., 2018; Manne-Goehler et al., 2019). This needs to be urgently addressed given the morbidity, mortality, and costs associated with diabetes (Glover et al., 2012; Glezeva et al., 2015; Low Wang et al., 2016; Atun et al., 2017; Adibe et al., 2018; Lewis et al., 2018; Pheiffer et al., 2018; Rwegerera et al., 2018; Mapa-Tassou et al., 2019). Eighty percent of early deaths due to non-communicable diseases (NCDs) including diabetes and cardiovascular diseases (CVD) currently occur in lower and middle income countries (LMICs), with the morbidity and mortality of diabetes and other NCDs such as CVD likely to be greater than communicable disease by 2025 (Peer et al., 2014; Renzaho, 2015; Issaka et al., 2018). Overall, SSA currently has the highest rate of morbidity and mortality associated with diabetes world-wide (Pastakia et al., 2017). In South Africa for instance as a result of changes in peoples' diet and lifestyle, diabetes has moved from being the fifth leading underlying cause of death in 2013 to now the second most common cause, representing 5.5% of all recorded deaths (Statistics South Africa, 2016; Moosa et al., 2019). T2DM also results in a lower health status and quality of life (Fasanmade and Dagogo-Jack, 2015; da Mata et al., 2016; Adibe et al., 2018; Rwegerera et al., 2018), with high rates of sight threatening retinopathy among diabetic patients in Africa (Glover et al., 2012; Jingi et al., 2015; Cairncross et al., 2017; Pastakia et al., 2017; Lewis et al., 2018) as well as nephropathy (Thomas et al., 2016; Wagnew et al., 2018) and neuropathy (Awadalla et al., 2017; Sun et al., 2020). Overall, populations of African origin appear to have the highest prevalence of microvascular complications of diabetes exacerbated by frequent high blood pressure and inappropriate diabetes control among the diabetes population along with challenges of access to appropriate care (Mbanya and Sobngwi, 2003; Mbanya et al., 2010).

**Table 1:** Synopsis of ongoing national and local activities to improve the management of patients with T2DM in sub-Saharan Africa.

Country	Activities
Botswana	As part of Botswana National Multisectoral Strategy for the Prevention and Control of Non-Communicable Diseases 2017–2022 a number of activities are in place including (Botswana National Multisectoral Strategy for the Prevention and Control of Non-Communicable Diseases, 2017): Disseminating information on NCD prevention including T2DM regularly in various media outlets Training and deploying community health agents (e.g., health education assistants) to conduct community outreach awareness and screening activities All schools to integrate NCD education in their health promotion curriculum Promoting access to healthy food, including at schools (taxation on imports of unhealthy foods, regulations on food provided at schools or packed from home in both private and public schools) Promoting physical activity at the workplace, at schools, and recreationally The Diabetes Association of Botswana collaborates with various organizations/companies to conduct staff wellness activities; with services including screening for diabetes and education on physical activity and healthy eating (Diabetes Association of Botswana, 2019) Establishment of diabetes clinics in different parts of the country as well as regular training of healthcare staff on diabetes management. Through the NCD unit at the Ministry of Health, education sessions on diabetes management using the primary health care guidelines—hypertension and diabetes (Tsima et al., 2016) Annual screening for diabetes mellitus across the country on World Diabetes Commemoration day Training of community health care professionals who are already involved in HIV care on diabetes management and education given the complexities involved including increased weight gain with treatments for HIV Increasing instigation of quality of care audits at different time intervals
Cameroon	There are currently no ongoing national and local activities to improve the management of patients with T2DM in Cameroon However, there are ongoing initiatives entitled “Cameroon National Diabetes and Hypertension Programme”—WDF16-1429 led by Prof Jean Claude Mbanya which aims at improving diagnosis and management as well as the prevention of diabetes and its complications in two regions of the country (North West and Centre) (World Diabetes Foundation, 2018) The key components of the program include: Building capacity to improve diabetes and hypertension care at both the national and district level Strengthening the administrative and monitoring capability of the Cameroon Ministry of Health Improving the surveillance systems for NCDs in the country Implementing campaigns among the population to raise awareness at the community level and among the media The outcomes of any initiatives will be reported in the future
Ghana	A Non-communicable Diseases Control Programme (NCDCP) was established by the Ministry of Health of Ghana in 1992 to respond to the growing burden of NCDs coordinating the national response to NCDs, working in partnership with other departments within the health sector, other ministries, NGOs, and civil society organizations (Bosu, 2012) In 1993, the NCDCP described general strategies for the prevention and control of chronic NCDs as well as disease-specific strategies, proposing a two-phase implementation of the program, from January 1994 to December 1998 and from January 1999 to December 2004, with specified targets for each phase (Sackey, 1993; Bosu, 2012). In 1994, the MoH identified the development of more effective and efficient systems for the surveillance, prevention, and control of non-communicable diseases as one of the main strategies to achieve its health service targets by the year 2008 (Bosu, 2012) In 1998, another strategy paper was prepared with the view to document the burden of the problem, identify the risk factors, and design the most appropriate intervention packages relevant to the Ghanaian situation (Bosu, 2012). In 2006–2007, strategic frameworks for the control of the major NCDs were developed with increasing levels of obesity and CV diseases in the Ghanaian population (Owiredu et al., 2008; Bosu, 2012) In June 2012, the Ministry of Health in Ghana launched its National Policy for the Prevention and Control of NCDs spanning 2012 to 2018 including issues of primary prevention and health system strengthening (Ministry of Health Ghana, 2012). While the National Health Insurance Scheme (NHIS) in Ghana does not reimburse healthcare facilities for secondary prevention in terms of routine screening services for patients with T2DM, NHIS covers tertiary care of DM patients in terms of their management. The facilities provide services including inpatient and outpatient services together with oral hypoglycemic drugs and insulins. All regional hospitals in the 10 administrative regions in Ghana and some selected district hospitals providing primary care have specialized DM clinics providing comprehensive care. These are either open for access in the entire week or have selected consulting days in the week More recently: There is also a new requirement by the Ghana Medical and Dental Council for doctors to accumulate credit points for re-licensing every year including updated information on the treatment of NCDs There is also a current initiative to improve on the clinical skills of pharmacists through the provision of the doctor of pharmacy (PharmD) program in all public universities as a first degree in pharmacy and also specialist pharmacist training at the both the Ghana College of Pharmacists and West African Postgraduate College of Pharmacist to improve on the quality of pharmaceutical care provided by pharmacists in both community and hospital pharmacies, which inlcludes patients with NCDs such as T2DM
Lesotho	A number of activities are ongoing including (World Health Organisation, 2016): Operational policies and strategic action plans for patients with diabetes including addressing key issues such as physical inactivity Ongoing developments surrounding guidelines and protocols of care for patients with diabetes Strategies to enhance the referral of patients from primary care to more specialized services when pertinent to help reduce future complications
Kenya	There are a number of strategies ongoing in Kenya to improve the management of patients with T2DM. These include: Implementation of the non-communicable disease 2015–2020 strategy (MoH. Republic of Kenya Ministry of Health), which includes raising the priority of NCDs such as T2DM, promoting healthy lifestyles, promoting and conducting research to reduce NCDs, as well as strengthen monitoring and evaluation systems. This builds on the Kenya National Diabetes Strategy 2010–2015 (Ministry of Public Health and Sanitation Kenya, 2010) Incorporation of diabetes education into the National Health Policy enhanced by the provision of Educator Manuals (Republic of Kenya Ministry of Health and Sanitation, 2010) as well as education to patients and healthcare providers by Diabetes Kenya whose mission is to provide a high standard of diabetes education and care as well as implementing prevention programs (Diabetes Kenya). Diabetes Kenya also links people with diabetes to resources and supply centers at discounted rates as well as publishing a quarterly journal, booklets on diabetes care and diabetes brochures to help improve the care of patients with diabetes in Kenya. Promotion of diabetes self management supported by regular clinical follow-ups and the incorporation of diabetes screening in all medical camp activities particularly those targeting rural hard to reach areas. Joint collaboration between Familia Nawiri, a social venture program initiated in Kenya by Novartis, and several County Ministries of Health in partnership with the Centre for Research in Therapeutic Sciences at Strathmore University, Kenya, and the Swiss Tropical and Public Health Institute, Switzerland, to strengthen the government's community health strategy through training of community health workers and health promotion at the household level (Karinja et al., 2019). Enhanced health cover for outpatient and inpatient care through the expanded National Hospital Insurance Fund with most patients with diabetes in Kenya in the rural setting diagnosed in public facilities (Karinja et al., 2019), although others have found that most patients are diagnosed in hospitals or private facilities rather than public clinics or health centers where there is a greater supply of appropriate medicines (Wirtz et al., 2018). Improving access to care including medicines (Sandoz - A Novartis Division, 2015; Shannon et al., 2019) and improving the supply chain, with metformin and glibenclamide the most prescribed medicines for patients with diabetes in Kenya (Karinja et al., 2019). In addition, enhancing access and availability of medicines through Universal Health Coverage (UHC). Currently, there is typically low availability of medicines in the public sector. As a result, most patients currently purchase their medicines privately from community pharmacies and clinics largely out of pocket even though they are available free-of-charge in public clinics (Wirtz et al., 2018; Nzwili, 2018; WHO Africa, 2018). Enhancing capacity building through targeted specialist training in diabetology. Sanofi in partnership with the International Diabetes Federation (IDF), Kenya Diabetes Study Group (KDSG), and Diabetes Kenya, supported by the Ministry of Health, launched mid 2019 a nationwide on-line 3-month diabetes management training program initially targeting 2000 General Practitioners. The objective being to help bridge the gap in the management of diabetes in Kenya with only a small number of diabetes specialists currently available (Destin Africa, 2019; Osei-Yeboah et al., 2019). Boehringer Ingelheim with PharmAccess has also recently launched a mobile technology enabled program, “Tiba Yako” for hypertension and diabetes patients to empower them to become more aware of their disease, access care, and take charge of their diabetes *via* a mobile health wallet, M-TIBA. This helps and enables patients to manage their diabetes from their own home through digital tools (Boehringer Ingelheim, 2019). The Digital Diabetes Patient Support Program (PSP) was also recently launched in Nairobi, Kenya, by Sanofi Kenya in partnership with CheckUps Medical Center in 2019. The program branded SPEED (Sanofi Patient Enlightenment and Empowerment Drive) looks to promote safe and effective use of medicines especially for patients with T2DM with up to 45% of adult diabetic patients or more currently not adhering to their oral diabetic medication (Aptantech, 2019).
Kingdom of Eswatini (formerly Swaziland)	There are ongoing initiatives in the Kingdom of Eswatini to increase awareness of T2DM especially among women including the necessity to adopt changes in lifestyle (WHO Swaziland, 2017), with diabetes accounting for 24% of deaths in patients with NCDs admitted to hospital (Kingdom of Eswatini - Ministry of Health, 2017), an increase on previous years. These include the development of essential healthcare packages including patients with chronic diseases (Magagula, 2017). The National Prevention and Control of NCD 2017 report highlighted a number of activities to help reduce morbidity and mortality of patients with diabetes (Kingdom of Eswatini - Ministry of Health, 2017). These included: The development of the Educational Health Care Plan which states that screening for identification as well as for management of diabetes should be offered at all levels of care. Selected screening tests such as blood sugar levels as well as fundoscopy and some specialized treatments such as nutritional supplements should be offered at all levels with currently only 35% of centers offering screening services. Restructuring and upgrading of services including sufficient human resource to enable decentralization of services from national to regional and local centers. Undertaking research to more accurately assess current incidence and prevalence rates to align services to improve future care.
Namibia	**National Policy Framework for diabetes prevention and control in Namibia**. Key areas include: Establishment of the National NCD Programme in the Ministry of Health and Social Services under the mandate of the office of the Prime Minister. Government commitment to funding public health care including diabetes. Regular updates and reviews of STGs and essential medicines lists incorporating diabetes care. Publication of the national multi-sectoral strategic plan for prevention and control of non-communicable diseases (NCDs) in Namibia 2017/18–2021/22 (Ministry of Health and Social Services - Primary Health Care Directorate, Family Health Division, 2017). Nutrition guidelines for prevention and management of non-communicable diet related diseases (Ministry of Health and Social Services Namibia, 2013). **Universal Health Coverage for diabetes**. Key aspects include: Provision of diabetic care as close to the family as possible at nearly no cost in the public sector and ensuring cost-effective access to care through various medical aid schemes since there are concerns with issues such as attendance in primary care clinics and adherence to medicines if facilities are not close to families (Nashilongo et al., 2017). Establishment of specialist-run Diabetes Clinics in referral hospitals. Integration of diabetes care as part of primary health care delivery including regular screening in the communities and improved access to diabetes testing at the community healthcare level. National laboratory service through the Namibia Institute of Pathology to provide cost-effective testing for diabetes. **Special interest and advocacy groups on diabetes** Commemoration of Healthy Lifestyles Day aimed at raising public awareness on the prevention of NCDs including diabetes. Community advocacy and awareness on diabetes by several interest groups such as the Diabetes Association of Namibia, University of Namibia Diabetes Association and Namibia Diabetes Lifestyle Foundation (Diabetes Association of Namibia, 2019; Namibia Diabetes lifestyle Foundation, 2019). Collaborative operational and clinical research on diabetes prevention, treatment, and control. **National surveillance of non-communicable and communicable diseases:** Undertaking Namibia demographic health surveys (NDHS) to ascertain the current prevalence of diabetes and prediabetes as a basis for developing future programs to reduce prevalence rates especially T2DM (Adekanmbi et al., 2019). **Building sustainable capacity in the care and control of diabetes especially T2DM** Incorporating NCDs as part of training curricula of pre-service and in-service health professional training programs including Medicine, Pharmacy, and Nursing.
Nigeria	On-going joint efforts by The Diabetes Association of Nigeria and The Federal Ministry of Health to review the Guideline for the management of Diabetes Mellitus in Nigeria aimed at addressing current challenges as the last edition of the guideline was published in 2011. Efforts to strengthen the care of patients with diabetes at the PHC level through development of guidelines, training of medical officers, nurses, pharmacists, and community health workers at the PHC level and establishment of diabetes mellitus registries at local government levels. Pilot scheme in Lagos State, Nigeria, being sponsored by the State Government and Health Matters Inc. to improve diabetes care in poor urban communities in Lagos City among 35 PHC facilities located in hard-to-reach slum areas. The project will seek to increase awareness about diabetes through awareness and screening camps *via* radio campaigns (World Diabetes Foundation, 2018). Awareness and Advocacy programs organized nationally and locally by various interest groups and societies such as Diabetes Association of Nigeria (DAN) and The Endocrine and Metabolic Society of Nigeria (EMSON) especially during World Diabetes Days with educational materials produced and distributed. Creation and regular meetings of Patient Support Groups by The Diabetes Association of Nigeria (Diabetes Association of Nigeria, 2019). Periodic free health screening programs at community levels to identify undiagnosed cases of T2DM.
South Africa	Various initiatives are ongoing to improve the care of patients with T2DM throughout South Africa. These include the following: Publication of updated South African guidelines for the management of patients with T2DM (SEMDSA, 2017). Recent instigation of a tax on sugar (Arthur, 2018). Increasing patients' empowerment of their T2DM through counseling and patient diaries *via* pharmacists and others, i.e., improve diabetes self-management (SEMDSA, 2017; Rampamba et al., 2019). Instigation of free screening centers for patients with suspected diabetes. Follow–up of patients with diabetes and those at high risk in the public sector by outreach healthcare workers in rural areas (Morris-Paxton et al., 2018) Potential instigation of ward-based outreach teams (WBOTs) to improve access to PHC services including health promotion and disease prevention for patients with T2DM (Khuzwayo and Moshabela, 2017). However, it is recognized that the management of patients with T2DM in the public sector is under-resourced. This may change with the introduction of a National Health Insurance Bill for Universal Health Coverage which will facilitate and promote the provision of health services for the management, prevention and control of communicable and NCDs (Republic of South Africa, 2019). Ongoing initiatives by the Government to realize its vision of a country free from the burden of disease include a multi-sectoral national wellness campaign “Cheka Impilo,” which is a call to action for South Africans to move from a curative response to health to preventative approaches and the adoption of healthy lifestyles. The campaign was launched on commemoration of World AIDS Day on 1 December 2018, and provides national support for testing and treating people who have HIV, TB, sexually transmitted infections, and NCDs such as diabetes and hypertension, reinforcing the implementation of prevention strategies, linkages to care, management, treatment, and support (Mabuza, 2018). National Adherence Guidelines for Chronic Diseases (HIV, TB, and NCDs) launched in 2016 for phased implementation throughout the country. This includes standard operating procedures for the early implementation of interventions in a sequential manner to support linkage, adherence, and retention in care (National Department of Health, 2016a; National Department of Health, 2016b). Evaluation of the impact of five of these interventions is currently being undertaken in a sample of PHC facilities in four of the provinces in South Africa (Fox et al., 2018). Implementation of the Centralized Chronic Medication Dispensing and Distribution (CCMDD) program, to improve access to chronic medication for stable patients through pre-dispensing and delivery to a point closest to the patient (Meyer et al., 2017). The program comprises two components: CCMDD and Pick-up Points (PuPs). With these initiatives, PHCs are decongested and patients will no longer be required to travel long distances and wait several hours to collect their medication at healthcare facilities. Synchronised National Communication in Health (SyNCH), which is a web system designed to improve process flows and transparency of the CCMDD program. This is a first for the South African public healthcare system, providing local, real-time data that informs decision-making and guideline development. Perceived advantages of the system include ensuring compliance to STGs, reducing medication errors and prescription rejections through validations built into the system, monitoring medicine collection status, and promoting the rational prescribing of essential medicines. In terms of patient care, SyNCH facilitates patient adherence to treatment through the PuP interface and automatic notifications to healthcare facilities for follow-up, thereby reducing the time taken to identify non-adherent patients. HIV Testing Service currently incudes screening for HIV, TB, diabetes, hypertension, obesity, and cervical cancer, with this service being used for screening multi-morbid conditions. Gauteng Province has been implementing a policy for health, happiness, and wellness of its residents through a comprehensive health prevention and promotion program. “Cities Changing Diabetes” project currently being implemented in Johannesburg. This includes establishing a health service laboratory in a PHC clinic where smart protocols, smart technologies (such as point of care machines) and smart medicines, including insulin analogues where appropriate, are being used under the leadership of a multi-disciplinary team comprising specialists in internal medicine, public health medicine, social workers, pharmacists, nursing staffs, allied health care workers, and community health care workers The South African Diabetes Association also provides educational and other support services; however variable influence on policies compared with other countries Of current concern is that the South African Nursing Council does not recognize the role and qualification of Diabetes Nurse Educators. However, there are ongoing moves to have this qualification officially recognized to help improve patient care in the future. In the private sector, diabetes is typically managed in conjunction with managed care programs with variable effect. Medication formularies are in place to support care and reduce costs. In general, though, the Health Insurance companies seem more concerned with month-by-month costs of management rather than longer term outcomes. However, there are moves to upskill healthcare professionals in diabetes management and care in the private sector. This includes attendance at a 5-day intensive, advanced course in diabetes care, offered by the Centre for Diabetes & Endocrinology (CDE) Academy. To date (July 2019) over 1,700 medical professionals, over 1,000 nurses, over 450 dieticians, as well as more than 300 other health care professionals have attended this particular course. This includes a number of participants from neighboring Southern African Development Community (SADAC) countries. The CDE also promotes further training and education of healthcare professionals in diabetes management and care, such as an online Postgraduate Diploma in Diabetes and MSc Diabetes offered by the University of South Wales. To date (July 2019) 168 successful Postgraduate Diabetes Diplomas and 49 MSc's in Diabetes have graduated. In addition to the above formal qualifications, there is an initial 10-week online foundation course in diabetes offered by the University of South Wales, and promoted by the CDE in South Africa.
Sudan	Ambulatory centers working as multidisciplinary institutions specializing in diabetes care were recently established in Sudan with funding from The World Diabetes Foundation. The aims are (Khogali et al., 2018): Provision of specialized medical care for diabetic patients Raising the public awareness about diabetes mellitus and its complications Enhance specialized training of medical and paramedical staff working in diabetes management. Establishing the Diabetes Care Organization, which is one of the voluntary non-profit organizations under the Sudan Diabetes Federation with the aim of leading the provision of Diabetic Health Care services to communities across Sudan.
Zambia	Zambia has recently introduced a national health insurance scheme aimed at improving and addressing access to healthcare and financing gaps, especially for patients with NCDs in Zambia. This is important since while Zambia has national policies and treatment guidelines for DM, operational policies and action plans are still required to reduce the risk of T2DM including public health promotion and educational prevention strategies in primary healthcare especially for high risk groups e.g., older age, overweight, and obese (Bailey et al., 2016). The future care of patients with T2DM should be helped by the introduction of healthy lifestyle campaigns (led by and exemplified by the Country President) to promote regular exercise as a preventive measure among the citizens of Zambia. The Diabetes Association of Zambia is helping here. However, Zambia currently lacks a national diabetes registry affecting health system planning and decision-making.
Zimbabwe	Zimbabwe adopted the primary health care approach according to the Alma Ata Declaration. There is currently a structure in place within the Ministry of Health handling NCDs headed by a deputy director. The office of the deputy director reports to the director of epidemiology who subsequently reports to the director of preventive services. There are two main organizations in Zimbabwe very active in issues related to patients with T2DM. The Zimbabwe Diabetic Association (ZDA) consists of patients with diabetes with a mission to improve the physical and socio-economic welfare of patients with diabetes in Zimbabwe through regular promotion and other activities (Zimbabwe Diabetic Association, 2019). Alongside this, there is the recently formed Zimbabwe Endocrinology, Diabetes, and Metabolism Association (ZEDMA) in April 2018. The Ministry of Health in Zimbabwe in 2019 planned to conduct an NCD risk survey with WHO support, with the last survey (STEPS) conducted 2005 giving a prevalence of T2DM at 10.2%. This is because the MoH acknowledges that NCDs have been largely neglected in preference to HIV and TB. Training workshops on essential NCDs package including T2DM will be conducted starting in August 2019. The Essential Drug List of Zimbabwe (EDLIZ), including the management of Metabolic and Endocrine conditions, will also be published in 2019 version (last updated in 2015). Zimbabwe guidelines for diabetes management are currently largely contained in the EDLIZ.

NB: CV, cardiovascular disease; DM, Diabetes Mellitus; HIV, human immunodeficiency virus; MoH, Ministry of Health; NCD, Non-communicable disease; PHC, primary healthcare; TB, tuberculosis; T2DM, Type 2 Diabetes.

Prevalence rates of T2DM vary considerably among individual African countries exacerbated by high rates of undiagnosed cases of diabetes (Atun et al., 2017; Stokes et al., 2017; Pheiffer et al., 2018; Asmelash and Asmelash, 2019; Manne-Goehler et al., 2019;) (**Appendix 1**). Overall, it is estimated that up to 70% or more of cases of diabetes are currently undiagnosed in SSA (Mbanya et al., 2010; Fasanmade and Dagogo-Jack, 2015; Pastakia et al., 2017). This is a concern as diabetes and its associated comorbidities increase cardiovascular diseases (CVD), which are responsible for approximately 70% of diabetes-related deaths (Glezeva et al., 2015; Low Wang et al., 2016; Mwita et al., 2017; Sarfo et al., 2018). It is hoped that ongoing activities across Africa (**Table 1**) will start to address this especially with mortality rates enhanced by uncontrolled hypertension as well as lack of control of HbA1c and lipid levels, especially low density lipoproteins (LDL-C), coupled with inactivity (U.K. Prospective Diabetes Study Group, 1995; UK Prospective Diabetes Study Group, 1998; MRC/BHF Heart Protection, 2002; Wan et al., 2017). Currently, only a minority of patients with diabetes in Africa achieve optimal therapeutic targets (Sobngwi et al., 2012; Pinchevsky et al., 2015; Manne-Goehler et al., 2019), which is a real concern.

The economic impact of diabetes in Africa is also increasing with growing prevalence rates with US$9.5billion currently spent on diabetes in Africa (International Diabetes Federation, 2019). Atun et al. recently estimated that in 2015, the overall cost of diabetes in SSA was 1.2% of the gross domestic product (GDP), with approximately US$10·81 billion spent on direct medical costs with out-of-pocket expenditure likely to exceed 50% of overall health expenditure in many of the countries (Atun et al., 2017). In Nigeria, estimates suggest the annual direct costs of diabetes are up to US$1.639 billion per year in view of its population size and current prevalence rates, with the estimated monthly direct medical costs for patients with T2DM in Cameroon at US$148 per patient and in Nigeria varying between $262.22 to $400.52 per patient (Mapa-Tassou et al., 2019). Median monthly healthcare expenditure in Kenya in households with diabetes was US$100.00 of which US$7.00 was on medicines (Wirtz et al., 2018). The effect of NCDs in the work place is also of growing concern across countries including Kenya and South Africa (Rasmussen et al., 2016).

Diagnosis rates need to be improved to achieve the World Health Organization's (WHO) 25×25 Global Action Plan (2013) as well as Sustainable Development Goal (SDG) 3 to reduce premature deaths due to NCDs by a third by 2030 (Joseph et al., 2017; Roth et al., 2017). Concerns with rising rates of T2DM and its impact will persist unless addressed. This includes access to diagnostic and monitoring equipment and essential medicines in the first place (Mbanya et al., 2010; Piloya-Were et al., 2016; Kibirige et al., 2017) along with adequate diagnostic facilities (Manne-Goehler et al., 2019) given current concerns with diagnosis rates. The WHO in 2015 found that in Africa only 51% of the countries had metformin routinely available and only 40% insulin, well below the 80% target (Atun et al., 2017). This is a major concern especially with only metformin along with gliclazide and glibenclamide included in the WHO essential medicines list (EML) (World Health Organization, 2019). There have been concerns though with hypoglycemia with sulfonyl ureas (SUs) (Naidoo et al., 2014). However, this applies more to glibenclamide than other SUs including glipizide, glimepiride, and gliclazide as well as gliclazide modified release (MR) (Abrahamson, 2015; Colagiuri et al., 2018; Kalra et al., 2018). This is reflected in the current South African guidelines recommending that glibenclamide should no longer be prescribed, with for instance gliclazide MR replacing the more general term SUs (Naidoo et al., 2014). Having said this in Nigeria, glibenclamide is still on the national EML; however, chlorpropamide is still being prescribed by some practitioners (Erah and Eroje, 2013; Federal Ministry of Health, Nigeria, 2016). There have also been concerns recently with limited availability of insulin analogues when needed especially with the increasing availability of lower costs biosimilars. However in the first instance, soluble insulin injections as well as intermediate acting insulins need to be made routinely available to T2DM patients within public healthcare systems across Africa in accordance with the 2019 WHO EML before potentially funding any analogue (World Health Organization, 2019).

The availability of medicines to treat patients with T2DM should improve with ongoing programs across Africa, including South Africa, to improve access to medicines in patients with chronic diseases (Meyer et al., 2017) as well as ongoing programs to reduce the costs of medicines as a barrier to their use (Sandoz - A Novartis Division, 2015). However, more remains to be done with continual concerns regarding the availability of metformin and insulins across Africa. Efficient procurement of medicines is critical to ensure that patients with diabetes within public healthcare systems are able to obtain an uninterrupted supply of their medicines (Wirtz et al., 2016).

Newer oral medicines for patients with T2DM such as the DPP4 (dipeptidyl peptidase-4) and SGLT2 (sodium/glucose cotransporter 2) inhibitors have also been proposed (Naidoo et al., 2014; Imprialos et al., 2017; Bailey and Day, 2019; Li et al., 2019). However, there are major issues with affordability within public healthcare systems struggling to routinely make available metformin and basic insulins. In addition, a number of patients with T2DM across Africa are ketosis prone and this is difficult to manage if their renal function is not routinely monitored in primary healthcare centers (PHCs). Moreover, it is difficult to estimate the true prevalence of patients who are ketosis prone across Africa. Ketosis-prone diabetes is an “atypical” form of diabetes particularly prevalent in diabetic patients in SSA with characteristics of both type 1 and type 2 diabetes (Ahrén et al., 1988; Mbanya et al., 2010; Sjöholm, 2019), principally though ketosis-prone T2DM (Sjöholm, 2019). Published figures suggest a prevalence up to 15% of the diabetes population in Africa have ketosis-prone diabetes (Sobngwi et al., 2002; Mbanya et al., 2010; Sjöholm, 2019); however, this could be an under estimate. In addition, there are also concerns with available resources and co-payments within public healthcare systems in Africa leading to continued endorsement and listing of appropriate SUs and metformin rather than funding newer oral medicines such as the DPP4 and SGLT2 inhibitors, as well as concerns with inertia with physicians prescribing insulins rather than again funding newer more expensive oral anti-diabetic medicines (Davies et al., 2018; Mwita et al., 2019).

Alongside this, many African countries currently have inadequate facilities to manage both the microvascular and macrovascular complications of T2DM (Atun et al., 2017). This includes addressing sub-optimal management of patients with T2DM with currently only a minority of patients in SSA achieving glycemic control (Mwita et al., 2012; Sobngwi et al., 2012; Kibirige et al., 2014; Pinchevsky et al., 2015; Kibirige et al., 2017; Manne-Goehler et al., 2019). Glycemic control is improved though in specialist centers in SSA (Sobngwi et al., 2012; Mwita et al., 2019; Rwegerera et al., 2019). This reflects the fact that NCDs such as diabetes have been relatively neglected over the last decade across Africa in favor of infectious diseases including human immunodeficiency viruses (HIV), tuberculosis (TB), and malaria due to their burden (Atun et al., 2017; Pastakia et al., 2017; South African Lancet National Commission, 2019). However, this is beginning to change (**Table 1**). Diabetic populations of African origin appear to have the highest prevalence of microvascular complications exacerbated by frequent hypertension and inappropriate diabetes control along with challenges with access to appropriate care (Mbanya and Sobngwi, 2003; Mbanya et al., 2010). Treatment of hypertension is a particular concern among the Black African population. Thiazide diuretics and calcium channels blockers rather than angiotensin-converting-enzyme inhibitors (ACE inhibitors) are now considered as the most effective antihypertensives in the Black African population (Choukem et al., 2007; James et al., 2014; SEMDSA Type 2 Diabetes Guidelines Expert Committee, 2017). Consequently, while ACE inhibitors are indicated for patients with diabetes and proteinuria, they have a lesser effect in reducing blood pressure in the Black African population and their prescribing needs to be carefully managed (Mbui et al., 2017; Mwita et al., 2019). There is also typically low use of statins among diabetic patients in Africa, which again needs to be urgently addressed to reduce future CV complications (Sobngwi et al., 2012; Uloko et al., 2012; Mwita et al., 2019).

Overall, a number of key issues have been identified surrounding the management of patients with T2DM in Africa that need to be addressed alongside issues of diagnosis and medicine availability. These include access to care in the first place, which is typically among public sector PHC facilities across most of SSA (Atun et al., 2017). Access to physicians and medicines continues to be a challenge especially if this involves high patient co-payments and/or there are long distances and queues to see a healthcare professional, both of which have economic and emotional consequences for patients (Mendenhall and Norris, 2015; Dube et al., 2017; Nashilongo et al., 2017). The prohibitive costs of medicines in some LMICs, which can account for up to 60% or more of total healthcare costs, does affect continued medicine use including adherence for long terms conditions such as T2DM if much of this is out-of-pocket (Cameron et al., 2009; Mhlanga and Suleman, 2014; Ofori-Asenso and Agyeman, 2016; Ong et al., 2018). For instance in Nigeria, typically household savings and family support are needed to fund treatments for patients with T2DM, with a mean monthly expenditure of US$356 per patient (Okoronkwo et al., 2016) similar to other studies (Mapa-Tassou et al., 2019). In addition, for patients with diabetes in Nigeria, the costs of medicines can account for over 70% of total direct medical costs (Fadare et al., 2015). Greater use of generic medicines could help reduce these costs (Akunne et al., 2016); however, there are issues with the quality of generics in Nigeria which needs to be addressed (Fadare et al., 2016).

Patient knowledge regarding diabetes and its management, including self-management, can also be poor among patients with T2DM in Africa (Mogre et al., 2017; Bonger et al., 2018; Stephani et al., 2018; Moosa et al., 2019). This is exacerbated by low levels of education among patients attending PHC facilities in SSA with CVD including T2DM and hypertension; however, this is not universal (Moosa et al., 2015; Kassahun et al., 2016; Nashilongo et al., 2017; Rampamba et al., 2017; Mufunda et al., 2018; Chang et al., 2019; Moosa et al., 2019; Niguse et al., 2019). In addition, there are concerns that being overweight and obese is still seen as a sign of affluence in a number of African communities, although changing, and appreciable weight loss still has the stigma of HIV and acquired immune deficiency syndrome (AIDS) associated with it. This is important especially in countries where there are high prevalence rates of HIV as well as high rates of patients with both HIV and T2DM as seen for instance in Botswana compared with high income countries (Hatsu et al., 2009; Rankgoane-Pono et al., 2018). Treatment, including adherence to agreed guidelines for patients with T2DM, and subsequent patient adherence to prescribed medicines, are also generally sub-optimal in LMICs including Africa (Igbojiaku et al., 2013; Awodele and Osuolale, 2015; Fadare et al., 2015; Moosa et al., 2015; Sapkota et al., 2015; Elsous et al., 2017; Abate, 2019; Moosa et al., 2019). A number of factors appear responsible for poor control of T2DM including the age of patients, their lack of perception of the consequences of diabetes, forgetfulness regarding taking medication, adverse effects of the prescribed medicines, living in rural areas, affordability, and issues of motivation including family support (Adegbola et al., 2016; Waari et al., 2018; Abate, 2019; Dedefo et al., 2019; Mwita et al., 2019; Rwegerera et al., 2019). There are also concerns with the concomitant management of hypertension and hypercholesterolemia along with controlling HbA1c in patients with T2DM in Africa (Gudina et al., 2011; Sobngwi et al., 2012; Mwita et al., 2019). Other potential factors impacting on key issues such as adherence include concomitant co-morbidities such as infectious diseases appreciably increasing the pill burden and regimen complexity (Libby et al., 2013). Treatments for HIV can themselves also lead to weight gain and obesity further complicating the management of T2DM patients with HIV (Bailey et al., 2016; Pastakia et al., 2017; Kumar and Samaras, 2018; Coetzee et al., 2019; Venter et al., 2019). A study undertaken in an HIV clinic in South Africa found the metabolic syndrome was seen in 20% of cases within 1 year after initiation of anti-retroviral treatment (ART) despite the young age of patients, presumably as a consequence of ART exposure (Julius et al., 2011). Similarly, diabetes increases the chances of patients getting TB nearly threefold (Pastakia et al., 2017). Consequently, T2DM patients with infectious disease co-morbidities must be carefully managed.

There have been a number of initiatives across Africa to improve identification, prevention, and management of T2DM to reduce subsequent morbidity, mortality, and costs. These include national strategies (Ministry of Public Health and Sanitation Kenya, 2010; Ministry of Health Ghana, 2012; National Department of Health, 2016a; National Department of Health, 2016b; Federal Ministry of Health, SIDCAIN, World Diabetes Foundation, 2017; Kingdom of Eswatini - Ministry of Health, 2017; Ministry of Health and Social Services Namibia, 2017; Mukanu et al., 2017) as well as the development of contextualized guidelines combined with research to demonstrate the need for strategies to improve guideline adherence and to assess their impact in practice (Igbojiaku et al., 2013; Mukanu et al., 2017; Pastakia et al., 2017; SEMDSA, 2017). There have also been strategies to improve access and availability of medicines (Sandoz - A Novartis Division, 2015; Meyer et al., 2017; Nuche-Berenguer and Kupfer, 2018; Shannon et al., 2019; Rockers et al., 2019), as well as strategies to improve the monitoring and follow-up of patients including advice on the doses of medicines prescribed (Pastakia et al., 2015; Morris-Paxton et al., 2018; Chang et al., 2019). However, such activities are not universal across Africa. Alongside this, there have been initiatives among African countries surrounding patient education and empowerment, along with support mechanisms such as the CCMDD initiative in South Africa, to improve access to treatment as well as adherence to suggested lifestyle changes and prescribed medicines (Ovbiagele, 2015; Kassahun et al., 2016; Jaam et al., 2017; Meyer et al., 2017; Amadi et al., 2018; Gathu et al., 2018; Mufunda et al., 2018; Stephani et al., 2018; Manne-Goehler et al., 2019; Rampamba et al., 2019). However, only a limited number of papers have been published assessing the impact of different initiatives in these patients (Sapkota et al., 2015; Gathu et al., 2018; Rampamba et al., 2019). We are also seeing the rise in mobile technologies to improve care including adherence to medicines (Farmer et al., 2019; Opoku et al., 2019), which will be one of the subjects for future research projects across Africa. Typically, multiple interventions are required to improve the care of patients with T2DM (Sapkota et al., 2015; Rockers et al., 2019). However, resources to expand such interventions to derive maximum benefits have to be balanced against ongoing needs in other high priority health conditions (Renzaho, 2015).

Consequently, the principal objective of this paper is to document and debate ongoing challenges and activities to improve the care of patients with T2DM in Africa especially around issues of adherence to changing lifestyles and prescribed medicines. This includes appraising key issues associated with co-morbidities with infectious diseases such as HIV and the resultant implications. There have been a number of systematic reviews looking at key issues surrounding the management of patients with diabetes in Africa including initiatives to strengthen healthcare systems (Brouwer et al., 2015; Atun et al., 2017; Hill et al., 2017; Issaka et al., 2018; Jaam et al., 2017; Nuche-Berenguer and Kupfer, 2018; Ong et al., 2018; Owolabi et al., 2018; Stephani et al., 2018; Wagnew et al., 2018; Asmelash and Asmelash, 2019; Manne-Goehler et al., 2019). We are also aware of the recent NCD research conference for Africa to share evidence and identifying research priorities (Juma et al., 2019). However to date, there have only been a limited number of publications that have comprehensively focused on all aspects of care covering diagnosis, prevention, education, and treatment including access to appropriate medicines for patients with T2DM and subsequent adherence rates. There have also been only a limited number of publications assessing the influence and impact of different interventions to improve the care of patients with T2DM across Africa. In view of this, we sought to document ongoing activities across Africa to improve the care of patients with T2DM as well as contextualize any findings from a wide range of co-authors from across Africa and wider to provide future guidance. This does not included issues of counterfeit medicines as this is outside the scope of this paper (Kawamura, 2011; Alghannam et al., 2014).

We have specifically chosen T2DM as this accounts for up to 95% of patients with diabetes in SSA (Fasanmade and Dagogo-Jack, 2015). In view of this, we believe our findings and subsequent implications should be of interest to key stakeholders across Africa and wider to help further improve the care of patients with T2DM building on current efforts. We will be monitoring these developments in the future.

## Our Approach

We did not perform a systematic review since there have been an appreciable number of recent publications, including systematic reviews, discussing ongoing research in this area and its implications including current prevalence rates, health system concerns, as well as possible policies and ways to improve future care (Brouwer et al., 2015; Agyemang et al., 2016; Hill et al., 2017; Jaam et al., 2017; Pastakia et al., 2017; Issaka et al., 2018; Nuche-Berenguer and Kupfer, 2018; Owolabi et al., 2018; Stephani et al., 2018; Wagnew et al., 2018; Asmelash and Asmelash, 2019; Farmer et al., 2019; Manne-Goehler et al., 2019; Mapa-Tassou et al., 2019). In addition, a number of the references, especially regarding ongoing national activities, are typically only available on the Internet as some of these have only just been launched. It is too early to assess the influence and impact of such national activities on key patient parameters as well as suggested activities to address key elements in the care provided. As mentioned, there have only been a limited number of publications to date in this area and only a limited number of studies including Sapkota et al. from LMICs (Sapkota et al., 2015; Gathu et al., 2018; Rampamba et al., 2019).

To address this knowledge and policy gap, we used senior level personnel from governments and their advisers, clinicians, academia, rational medicine use advisers, health technology assessment (HTA) personnel, as well as patient organization personnel, to suggest future activities for all key stakeholder groups across Africa to improve the future care of patients with T2DM. This included both short- and long-term initiatives, with the advice given based on relevant publications known to the co-authors coupled with their considerable knowledge of ongoing activities in their own countries. This builds on policy gaps and other recent papers to help improve the care of patients with T2DM across Africa (Atun et al., 2017; Pastakia et al., 2017 Owolabi et al., 2018; Manne-Goehler et al., 2019). The co-authors came from a wide range of backgrounds and countries in terms of their geography, population size, GDP per capita, as well as progress toward universal healthcare. We have successfully used this approach before to stimulate debate in other priority healthcare areas and situations to provide future guidance (Godman et al., 2010; Godman et al., 2014a; Godman et al., 2014b; Campbell et al., 2015; Godman et al., 2015; Ermisch et al., 2016; Bochenek et al., 2017; Moorkens et al., 2017; Godman et al., 2018; Vella Bonanno et al., 2019; Godman et al., 2020).

We did not split the African countries into low- or middle-income countries as the burden of T2DM and its implications is growing across all of Africa. Consequently, countries can learn from each other. We also did not systematically review each paper for its quality using well-known scales such as the Newcastle-Ottawa scale or the Cochrane risk of bias tool as our emphasis was on contextualizing the findings rather than performing a systematic review for the reasons given (Marra et al., 2016; Ong et al., 2018; Almeida et al., 2018; da Silva et al., 2018; Saleem et al., 2019). In addition, we did not review each paper to assess whether the presence of diabetes, especially T2DM, had been defined according to WHO and other internationally recognized diagnostic criteria in view of the objectives of this paper (World Health Organisation, 2006; Manne-Goehler et al., 2019).

## Ongoing Activities and the Implications

Ongoing initiatives and activities among a range of African countries to improve the identification and management of patients with T2DM will be described first before debating potential approaches among all key stakeholder groups to improve future identification and management of patients with T2DM across Africa. This includes the cascade of treating patients with T2DM starting with glucose testing, diagnosis, lifestyle advice, as well as the prescribing of medicines and counseling for adherence to prescribed medicines (Manne-Goehler et al., 2019). We are aware for instance that most guidelines on the management of patients with T2DM in LMICs including African countries are inadequate in terms of their applicability, clarity, and active plans for dissemination (Owolabi et al., 2018). We are also aware that national guidelines when produced are not always readily available in primary and community healthcare centers or routinely consulted (Mashalla et al., 2016; Matsitse et al., 2017).

As mentioned, there will be a particular focus on strategies to improve access to medicines and subsequent adherence rates to prescribed medicines based on the combined experiences of the co-authors and the published literature.

### Ongoing Initiatives and Activities Among African Countries

**Table 1** contains details of a range of activities being undertaken among SSA countries to improve the management of patients with T2DM.

### Suggested Activities Among All Key Stakeholder Groups to Improve Future Management of Patients With T2DM

We have based suggested activities and initiatives that still need to be undertaken among key stakeholder groups across Africa to improve the cascade of patient management (Manne-Goehler et al., 2019), and subsequently reduce future morbidity, mortality, and costs due to T2DM and its complications, principally on the experiences of the co-authors in the absence of published data. These are contained in **Boxes 1**–**5** and build on **Table 1**.

Box 1 **Suggested activities among national governments and authorities.**

**A) Short term**

- Prioritize the screening for diabetes at all health care facilities in the country including those with diabetes alongside infectious diseases such as TB (Segafredo et al., 2019). This includes providing the basic tools and technologies to identify at risk patients and to monitor their progress—including providing BP monitors in all clinics alongside glucose monitoring systems, with the provision of self-monitoring systems important for patients on insulin.
- Improve the routine availability of medicines in the public sector to treat patients with T2DM including addressing concerns with the current lack of metformin, SUs, or insulins when this occurs. This includes ensuring the availability and use of low cost quality medicines (generics) to reduce patient co-payments where applicable. Initiatives could include improved supply chain management incorporating better stock control and forecasting abilities—building on initiatives in South Africa such as the Central Chronic Medication Dispensing and Distribution (CCMDD) initiative (Meyer et al., 2017)—as well as improved regulatory controls limiting the availability of poor quality generics where this is a concern.
- Introduce or enhance the implementation of programs including educational programs among T2DM patients to improve their lifestyles in association with other groups such as patient associations and other relevant NGOs.
- Review and improve where pertinent the training of healthcare professionals in the management of patients with T2DM in both undergraduate and postgraduate programs to enhance the future quality of care. This can include instigating CPD activities, and builds on ongoing programs in Ghana, South Africa, and wider.
- Instigate/refine training programs at both undergraduate and postgraduate levels to improve the care of patients with T2DM. This can include developing and implementing CPD programs building on initiatives in Ghana to upskill their knowledge—including understanding of the different oral medicines to treat T2DM and insulin preparations as well as their role and place especially in patients with various co-morbidities.
- Instigate policies to enhance adherence to prescribed medicines and dietary modifications building on ongoing initiatives across SSA including Kenya and South Africa as well as learnings from other NCDs. This involves improved understanding of key factors influencing adherence including the level of education, family and support systems, and funding issues, which is critical to enhance the future care of T2DM patients. It is likely mobile technologies will play an increasing role.
- Instigate training and other measures to improve the detection and treatment of T2DM-related causes of blindness such as diabetic retinopathy as well as causes of renal complications including preventative measures along with general measures to improve BP control (Chawla et al., 2010; Cappuccio and Miller, 2016).

**B) Longer term**

- ***Leadership and governance***
- Greater prioritization of NCDs especially T2DM with its implications for morbidity, mortality, and costs. As part of this, instigate activities to develop or refine national strategies to improve the care of patients with T2DM, building on current activities among SSA countries (**Table 1**). This can include implementing national NCD strategies with their emphasis on identification, healthy lifestyle changes (physical activity, healthy eating, limiting processed foods, and weight management—including addressing concerns with any stigma/negative perceptions associated with weight loss) along with adequate treatment of patients with T2DM based on agreed national guidelines (cognizant of local co-morbidities including infectious diseases among the different African countries).
- Conduct screening for NCDs including T2DM among at risk individuals including those with a family history of diabetes, overweight or obesity at schools, workplaces, churches, and shopping centers as well as in PHC facilities.
- ***Health workforce***
- Strengthen health care systems including ambulatory care systems as well as policies and initiatives to better identify and manage patients with T2DM. This can include making better use of healthcare professionals and community health workers in primary/community health clinics as well as community pharmacists to help with education and medication adherence (**Boxes 3** and **4**).
- Seek to address shortages of physicians where applicable including training and utilizing of other healthcare professionals such as clinical associates (or clinical officers), nurses, and community pharmacists to help manage patients with T2DM. This can also include instigating home visits by healthcare professionals such as dieticians, diabetic nurse educators, and podiatrists, to improve the care of patients; however, instilling that complex cases must be rapidly referred to specialists.
- Task-shifting through utilization of mid-level workers (such as pharmacists, physiotherapists and podiatrists) to support the clinical management of NCDs. As a result, enhance the multidisciplinary approach to the management of patients with T2DM throughout the healthcare system.
- Undertake multidisciplinary research at local, regional, and national settings to improve the future care of patents with T2DM including adherence to medicines, and seek to instigate any findings.
- ***Financing***
- Accelerate the introduction of universal healthcare in the public sector where patient co-payments are currently an issue to improve the care of patients with T2DM.
- ***Improved efficiency***
- Strengthen HTA capabilities especially when Ministries of Health are confronted with a number of potential initiatives coupled with limited resources, building on ongoing initiatives across sub-Saharan Africa including Ghana, Kenya, and South Africa (Hernandez-Villafuerte et al., 2016; Mueller et al., 2017; Hollingworth et al., 2018; Southern African Health Technology Assessment Society (SAHTAS), 2019).
- Address the social and cultural determinants of health in patients with T2DM through leadership at a national level if not already underway—building on initiatives in Botswana, Ghana, Kenya, the Kingdom of Eswatini, Namibia, and South Africa to prevent or manage the risk factors associated with T2DM through greater stakeholder collaboration (MoH. Republic of Kenya Ministry of Health; National Department of Health South Africa, 2013; Ministry of Health and Social Services Namibia, 2017; Kingdom of Eswatini - Ministry of Health, 2017; Botswana National Multisectoral Strategy for the Prevention and Control of Non-Communicable Diseases, 2017; South African Lancet National Commission, 2019).
- Disseminate NCD information including T2DM regularly in national political forums, e.g., public speeches and in parliamentary debates.
- Strengthen the monitoring and evaluation of the management and treatment of T2DM to ensure that efforts are in line with the burden of NCDs. This includes regular auditing of the management of patients with T2DM against agreed national guidelines and seek to introduce quality indicators where concerns. Quality indicators can include HbA1c, BP, and lipid level goals developed with the help of all key stakeholders using robust methodologies (Campbell et al., 2011; Campbell et al., 2015).
- ***Research***
- Continue to research methods to improve medication and dietary adherence including counseling, addressing concerns with processed foods and addressing patients' fears and beliefs including weight loss. In addition, researching potentially patient diaries and mobile messaging services as well as measures to enhance patient satisfaction with healthcare services (Habte et al., 2017; Waari et al., 2018; Moosa et al., 2019; Farmer et al., 2019; Rampamba et al., 2019). Subsequently, seek to instigate such findings.
- Fund ongoing research around diabetic phenotypes among the African population compared with other continents and its impact on key issues such as insulin resistance to improve future care (Pastakia et al., 2017; Kibirige et al., 2019).

NB: BP, Blood Pressure; CPD, Continual Professional Development; HTA, Health Technology Assessment; NCD, Non-communicable disease; NGOs, Non-governmental organizations; PHCs, Primary Healthcare Centres; SSA, sub-Saharan Africa; SUs, Sulphonylureas; TB, Tuberculosis; T2DM, Type 2 Diabetes.

Box 2 **Suggested activities among physicians (hospital and ambulatory care).**

**A) Short term**

- Instigate/refine training programs at both undergraduate and postgraduate levels to improve the care of patients with T2DM.
- This can include developing and implementing CPD programs building on initiatives in Ghana as well as programs to enhance timely diagnosis, management and follow-up of patients with T2DM including the management of patients with T2DM with HIV and TB.
- Instigate in-service training where pertinent including any updated guidelines for new medicines.
- Take part in Pharmaceutical (Drug) and Therapeutic Committees and other fora to ensure possible treatment decisions are in line with national guidelines and essential medicine lists, and evidence–based especially where co-morbidities.
- Ensure prompt referral of patients (ambulatory/primary care) to specialized centers where these exist and where necessary—including complex patients with multiple co-morbidities.
- Formulate care plans for patients and monitor prescribing (audit) against agreed guidance, especially those with co-morbidities including infectious diseases with rapid referral to specialist centers for more complex patients including those with multiple co-morbidities.
- Communicate with patients the importance of adherence to medicines, and seek to monitor this. This can be through diaries and other mechanisms including the use of mobile technologies.

**B) Longer term**

- Actively become involved in future research to improve the management of patients with T2DM across Africa given the high rate of co-morbidities especially infectious diseases in SSA compared with other continents as well as positive views toward overweight and obesity among populations especially in SSA.

NB: CPD, continuous professional development; HIV, human immunodeficiency virus; SSA, sub-Saharan Africa; TB, tuberculosis; T2DM,Type 2 Diabetes.

Box 3 **Suggested activities among pharmacists (hospital and ambulatory care)**

**A) Short term**

- Extend the role of pharmacists in hospitals and in the community to help improve the care of patients with T2DM. This can include counseling on issues of medication adherence, potential side-effects of medicines, and dispelling myths especially with community pharmacists often the first point of contact for patients with ailments in Africa.
- Encourage pharmacists to join national diabetes groups (such as ZEDMA in Zimbabwe) to help debate key issues regarding the use of medicines to treat patients with T2DM.
- Pharmacists as a vital part of the medicine supply chain need to instigate measures to reduce out-of-stock situations for essential medicines to treat patients with T2DM in public health facilities. The objective being to reduce reliance on private pharmacies especially where co-payments are an issue.
- Pharmacists should also monitor medicine availability in hospitals as part of DTC activities to help reduce out-of-stock situations, and work with physicians and others to instigate alternative medicines should the need arise.
- Pharmacists should also seek to encourage the use of generic medicines where possible to reduce patient co-payments where pertinent as well as healthcare costs.

**B) Longer term**

- Pharmacists need to become actively involved with auditing medicine use and providing support and other services to improve medication adherence. These can include mobile alerts, diaries, storyboards, and general information to enhance adherence, and can be part of DTC activities in hospitals as well as part of PHC activities in ambulatory care.
- Take part in research regarding factors impacting on medication adherence and possible ways to address this within their own health service, and seek to actively address and implement key findings.

NB: DTC, Drug and Therapeutic Committee; PHC, Primary Healthcare Centre; SSA, sub-Saharan Africa; T2DM, Type 2 diabetes.

Box 4 **Suggested activities among other healthcare professionals in ambulatory and hospital care including nurse practitioners**

**A) Short term**

- Use nurse practitioners and others to help screen for T2DM in the community especially at risk groups **(Box 1)**
- Work with other key stakeholders to provide accurate information to patients on the management of their condition including self-care to help prevent complications from occurring

**B) Longer term**

- Become involved with developing minimal standards of care for patients with T2DM in primary/community healthcare centers and subsequently monitoring the management of patients against agreed standards. This can include lifestyle changes and adherence to medicines as well as identifying acute and chronic complications for rapid referral
- Actively seek ways to provide support to patients and their caregivers to take control of their own health and to enhance adherence to lifestyle advice and any medicines prescribed
- Actively seek to improve the knowledge of medicines and self-management practices among fellow nurses and other community health workers regarding the care of patients with T2DM to enhance task shifting efforts at the primary/community level—ultimately helping to reduce time spent on long queues and the implications on key issues such as adherence. Concurrently with this, rapidly refer more complex patients to specialists given ongoing concerns
- Provide evidence–based care especially regarding complications of diabetes such as ulcers
- Take part in research initiatives to improve the future care of patients with T2DM, and seek to instigate the outcome of any research activities.

NB: T2DM, Type 2 diabetes.

Box 5 **Suggested activities among patients/support services.**

**A) Short term**

- Help communicate the importance of detection and management of T2DM including lifestyle changes and adherence to medicines to help reduce the morbidity and mortality of T2DM. This builds on progressive activities of a number of diabetes patient groups in SSA (**Table 1**) and is especially important where there are limited facilities to manage the complications of diabetes including renal complications, retinopathy and diabetic ulceration.
- Help to instigate communication and other programs to encourage patients to join national Diabetic Associations as well as generally encouraging patients and their carers to take part in peer education and support groups.
- Help provide easy to understand guidance to patients to help with their understanding of their condition as well as improve the management of their condition including lifestyle and medication advice. Such guidance should also help to dispel cultural and social stigmas associated with T2DM including those surrounding weight loss.
- Instigate activities encouraging recreational activities as well encouraging patients to ask questions when they are still unsure of their condition/instructions given and to seek prompt medical help when they experience changes in their condition.
- Seek generally to strengthen family support structures through education and socio-economic empowerment.
- Work with all key stakeholders to enhance their understanding of any concerns that patients and their support network may have regarding the management of T2DM and potential complications to better target educational and other activities.

**B) Longer term**

- Formulate and empower support groups among patients with T2DM such as adherence clubs to enhance the transfer of knowledge and self-management aspects among patients.
- Help longer term to develop innovative interventions tailored to the culture of patients in given African countries to enhance adherence to medication and lifestyle changes, such as storytelling, which have shown to be successful in other NCDs (Houston et al., 2011). This can increasingly be *via* mobile technologies and other support systems (Bonoto et al., 2017; Lewis et al., 2018; Farmer et al., 2019; Owolabi and Goon, 2019).
- Encourage greater involvement in the prevention of diabetes complications by self-monitoring of foot health and active participation in screening for eye, feet, and renal complications.
- Encourage key stakeholders to include patient support groups in future research activities and with the communication of their findings to improve future care.

NB: T2DM, Type 2 diabetes.

Suggested activities include increasing recognition of the burden of T2DM across Africa to raise its priority status. As a result, seek to appreciably reduce the number of patients across Africa currently not being diagnosed with T2DM including those with pre-diabetes. Concerns with the availability and use of medicines, especially those contained in the WHO EML, needs to be addressed including metformin. There are also concerns with poor adherence rates to prescribed medicines and prescribing guidelines, the routine availability of prescribed medicines, and the development of insulin resistance among diabetic patients. All these concerns need to be addressed to reduce future morbidity and mortality. The complexity of the management of patients with T2DM is enhanced if they have infectious diseases such as HIV and TB, which is appreciably more likely in SSA than high income countries where most guidelines emanate from. As a result, guidelines need be country and region specific taking into account genetic and other factors including patients with diabetes across Africa having low to normal BMIs, those with ketosis-prone T2DM, as well as appropriate management of those with co-morbidities including hypertension and infectious diseases. This is unlike the situation in many other continents.

Suggested activities will include those at governmental and national levels as well as among the different healthcare professional groups and patients to improve the future care of patients with T2DM. Potential initiatives to improve adherence rates to prescribed medicines will also be discussed given current concerns.

In addition, there is an ongoing need to improve HTA capabilities across Africa to help fully assess the effectiveness and cost-effectiveness of different approaches, including different treatment approaches, especially given limited available resources and potentially multiple possible initiatives. This can build on existing research across countries (Kriza et al., 2014; Hernandez-Villafuerte et al., 2016; Doherty et al., 2017; Mueller et al., 2017; Hollingworth et al., 2018; Juma et al., 2019) for evidence–based decision making.

## Discussion and Conclusion

We believe this is the first comprehensive review to collate ongoing and planned activities across Africa to improve the management of patients with T2DM as well as suggest potential future strategies especially around medicine management. As a result, enable African countries to compare and contrast ongoing activities as well as debate potential future activities while the evidence base grows regarding their potential influence on reducing future morbidity, mortality, and costs due to T2DM.

It is encouraging to see that an appreciable number of African countries are actively instigating programs to improve the care of patients with T2DM starting with improving diagnosis (**Table 1**), with countries such as Cameroon looking to instigate pilot activities starting in key regions. This is important with T2DM a growing problem across Africa with an estimated 19 million adults in Africa currently with diabetes and increasing (International Diabetes Federation, 2019), and recognizes the growing burden of NCDs across Africa, and we will be reporting on some of the findings in the future.

Key national activities in Africa (**Box 1**) include health system strengthening as well as improving the subsequent management of patients with T2DM including those with co-morbidities. This starts with the development or refinement of national action plans to enhance the care of patients with NCDs including those with T2DM. Initial activities include improved screening and diagnosis of high risk patients at a number of venues including schools, workplaces, churches, and shopping centers as well as in PHC facilities. This includes patients with pre-diabetes especially those with other cardiovascular diseases (Kharroubi and Darwish, 2015; Mbugua et al., 2017; Todowede and Sartorius, 2017) to reduce future morbidity, mortality, and costs. Treatment approaches comprise both pharmacological treatments to prevent cardiovascular complications as well as lifestyle changes including diet changes.

Access to medicines is especially important across Africa with for instance up to 75% of patients having difficulties with obtaining insulin (Manne-Goehler et al., 2019). The very least is the routine availability of metformin, appropriate SUs, and soluble and intermediate insulins as well as the routine availability of monitoring equipment for T2DM patients on insulin. The development of universal health care as well as the assistance of donors and commercial organizations can be beneficial here (Sandoz - A Novartis Division, 2015; Meyer et al., 2017; Shannon et al., 2019). Initiatives to encourage the use of generics where possible, obtain low prices for quality medicines as well as instigate effective distribution systems can also enhance access to effective medicines in both public and community health centers (Cameron et al., 2012; Kaplan et al., 2012; Meyer et al., 2017; Pastakia et al., 2017). The development and instigation of evidence–based guidelines taking account of local co-morbidities including HIV and TB as well as genetics will also be a key step towards improving the management of patients with T2DM across Africa, enhanced by the auditing of current prescribing patterns (Pastakia et al., 2017; Owolabi et al., 2018). Furthermore, key stakeholder involvement in the production of country guidelines is seen as important to enhance their utilization building on the experiences in other countries (Gustafsson et al., 2011; Bjorkhem-Bergman et al., 2013; Eriksen et al., 2017).

A key concern across Africa is the multi-morbidity among patients with T2DM including co-morbidity with HIV and TB. This adds to the burden of the disease, exacerbated by these infectious conditions. Consequently, country specific guidelines need to be cognizant of this with rapid referral systems in place for more complex cases given the improved management of patients in tertiary centers. Prescribers need to be cognizant of the potential for HIV treatments to exacerbate diabetes and also for diabetes to exacerbate TB. This needs to be emphasized during physician and healthcare professional training, followed up by continuous professional development and other activities.

Concerns with the pharmacological management of patients with T2DM include adherence to medicines, which is typically sub-standard, as well as insulin resistance. A number of studies have shown the importance of healthcare professionals such as pharmacists, nurse practitioners, and community health workers, improving subsequent adherence and outcomes (Dube et al., 2017; Ong et al., 2018), with more studies planned given current concerns. We envisage that innovative approaches such as the increasing use of mobile technologies will be adopted to enhance adherence to lifestyle changes and medicines, and we will be monitoring this development in the future (Opoku et al., 2019; Owolabi and Goon, 2019). Countries can also continue to learn from each other to improve future care.

Whatever new initiatives or treatment approaches are chosen, it is likely that countries will need to expand on their HTA capabilities to assist with rational choices given continuing pressure on resources. This is already happening in countries such as Ghana and South Africa, and will expand further. Potential choices for governments consist of enhancing the infrastructure including public/community healthcare centers with multiple personnel incorporating physicians, medical officers, pharmacists, nurses, and others, to aid identification and reduce the development of complications in patients with T2DM and associated hospitalization. In addition, seek ways to address key issues regarding the availability of medicines for the management of diabetes and its complications including reducing co-payments where these exist. Multiple approaches also appear needed to address poor adherence rates to prescribed medicines where these occur, which will be the subject of future research projects.

In conclusion, we believe our findings and subsequent implications, including suggested activities (**Boxes 1** to **5**) for all key stakeholder groups, should be of interest to key groups across Africa and wider to improve the future care of patients with T2DM, building on current efforts (**Table 1**). This is essential given rising rates of obesity in Africa with their subsequent impact on morbidity, mortality, and costs. We will be monitoring these developments in the future especially as more studies become available evaluating ongoing initiatives among the different African countries and the implications.

## Author Contributions

BG, JF, OO and JoM devised the concept of the paper. All authors subsequently contributed to its content and approved the final version.

## Conflict of Interest

LD is employed by the Centre for Diabetes & Endocrinology (Pty) Ltd.

The remaining authors declare that the research was conducted in the absence of any commercial or financial relationships that could be construed as a potential conflict of interest. However, a number of them are employed by national or regional governments in Ministries of Health or are advisers to them. In addition, advisers to the World Health Organisation.

## Appendix 1 Prevalence rates of pre-diabetes and diabetes among a range of African countries

**Table:**

Country	Prevalence rates
Republic of Benin	Prevalence rates including undiagnosed diabetes have been as high as 12.4% of the population although other authors have quoted considerably lower rates (Djrolo et al., 2015; Pastakia et al., 2017)
Botswana	Documented prevalence rates for diabetes range from 4.8 to 6% of adults, with prevalence rates rising with increasing rates of obesity in recent years (World Health Organization, 2018)
Cameroon	The prevalence of pre-diabetes is 7.1% (Bigna et al., 2018), with the prevalence of diabetes in adults in urban areas currently estimated at 6–8% of the population (World Diabetes Foundation, 2018) It is estimated that with as many as 80% of patients living with diabetes are currently undiagnosed (World Diabetes Foundation, 2018)
Democratic Republic of Congo (DRC)	There are an estimated 1.8 million (1.5–2.2 million) people with diabetes in DRC (SEMDSA, 2017)
Ethiopia	Up to 7% of the population have diabetes, with an appreciable number of patients unaware that they have this condition (Zekewos et al., 2018; Abate, 2019)
Ghana	A recent meta analysis suggested the overall prevalence of diabetes among adults was high at 6.46% of the population (Asamoah-Boaheng et al., 2019), with previous studies also documenting high rates of diabetes in Ghana (Ministry of Health Ghana, 2012; Bosu, 2012)
Kenya	The prevalence of diabetes including T2DM in patients in Kenya has been estimated at 3.3% and rising up to 4.5% by 2025, leading to the development of national strategies to reduce associated morbidity, mortality, and costs (Ministry of Public Health and Sanitation Kenya, 2010; Waari et al., 2018), while others have reported lower and higher rates (Pastakia et al., 2013; Mohamed et al., 2018) Having said this, Bastawrous *et al*. (2018) estimated the cumulative incidence of DM at 61·0 per 1,000 in people aged ≥50 years in Kenya (Bastawrous et al., 2017), with recent increases exacerbated by changes in diet and inactivity (Onyango and Onyango, 2018) Published prevalence rates in Kenya are likely to be under-estimates considering the number of patients with undiagnosed diabetes and pre-diabetes (Mohamed et al., 2018) The International Diabetes Federation current estimates that there are 460,000 people with diabetes in Kenya and likely to rise to 1.3 million within a generation unless addressed (Destin Africa, 2019)
Lesotho	The prevalence of diabetes is 6–4.5% males and 7.5% females (World Health Organisation, 2016)
Kingdom of Eswatini (formerly Swaziland)	The prevalence of diabetes in the Kingdom of Eswatini is currently estimated at 3.7% among the adult population, and predicted to rise substantially in the coming years unless key changes are made to the management of patients with diabetes in the country (Burn and Pons, 2015) Currently, patients with diabetes account for 9.5% of all outpatient visits in the Kingdom, with a greater prevalence among women (59%), and 17.4% of inpatients by admission (Kingdom of Eswatini - Ministry of Health, 2017)
Namibia	The prevalence of diabetes has been estimated at 5.1% and pre-diabetes at 6.8% (Adekanmbi et al., 2019) Ministry of Health figures suggest higher rates at 6% among women and 7% among men with 7% of women and 6% of men pre-diabetic (Ministry of Health and Social Services - Primary Health Care Directorate, Family Health Division, 2017), with rates higher in urban *versus* rural areas
Nigeria	The age-adjusted prevalence rates for T2DM among those aged between 20 and 79 years has increased from 2.0% in 1990 to 5.7% in 2015, and rising (Adeloye et al., 2017), with others reporting prevalence rates as high as 10% of the population (Ogbera and Ekpebegh, 2014; Amadi et al., 2018) A recent systematic review suggests an overall prevalence of 5.77%, highest in the south east (9.8%) and lowest in the north-east (3.8%) with urban dwelling, physical inactivity, and unhealthy diets seen as are important risk factors (Fasanmade and Dagogo-Jack, 2015; Uloko et al., 2018) However, it is estimated that up to 70–80% of patients with diabetes in Nigeria are either undiagnosed or untreated (Fasanmade and Dagogo-Jack, 2015), with Adeloye et al suggesting a pooled rate of 39.4% in their systematic review (Adeloye et al., 2017). This is important since Nigeria currently accounts for one fifth of all the cases of diabetes in sub-Saharan Africa (Fasanmade and Dagogo-Jack, 2015) Diabetes is also observed in up to a third of all admissions in medical wards adding to the burden (Fasanmade and Dagogo-Jack, 2015).
South Africa	Published prevalence rates for diabetes vary between 7.2% and 10.1% or higher of the population depending whether undiagnosed patients are included (Manyema et al., 2015; Bailey et al., 2016; Shen et al., 2016; Stokes et al., 2017; Morris-Paxton et al., 2018). Overall, it is estimated that 4 million South Africans currently have diabetes (1.2–4.6 million) (SEMDSA, 2017; Juma et al., 2019) However, based on the SANHANES data there is a higher prevalence of diabetes in South Africa (15.3%) should a high percentage of patients with diabetes actually be diagnosed and treated (Stokes et al., 2017; Basu et al., 2019) A recent report from Johannesburg found 66% obesity and overweight in a random population attending primary healthcare (PHC) clinics, with a 4.9% undiagnosed diabetes. All the cases, who were diagnosed are receiving care (100%) and of all the patients, who are receiving care, 31% of them were controlled. The prevalence of co-morbidities including family history, obesity, overweight, hypercholesterolemia, hypertension, and HIV were common The analysis of Hba1c results from National Health Laboratory Service (2017) found 16% of 1.6 million patients from public health facilities in Gauteng Province had Hba1c <7%
Sudan	The current prevalence of diabetes in Sudan is estimated at 8%; however, this is not evenly distributed over the country. Prevalence rates are higher in some Northern states, and higher in urban (19.1%) *versus* rural area (2.6%) (Noor et al., 2015; Elmadhoun et al., 2016). Individuals of Egyptian and mixed descent also have increased risk of getting T2DM (Eltom et al., 2018) Researchers attributed the increase in prevalence of diabetes in Sudan to the increase in obesity rates, increased sugar intake, lack of physical activity, and excess carbohydrate intake (Ali et al., 2017; Khalil et al., 2017; El-Sayed et al., 2018), with approximately 40% of the cases of T2DM attributable to obesity among the population in Sudan
Zambia	Prevalence rates for diabetes are estimated at 3.5% up to 5.35% of the population (Bailey et al., 2016; Mukanu et al., 2017)
Zimbabwe	Pooled prevalence rates suggest 5.7% of the population have diabetes and rising (Mutowo et al., 2015)
